# Using *Spirulina platensis* as a natural biocoagulant for polystyrene removal from aqueous medium: performance, optimization, and modeling

**DOI:** 10.1038/s41598-024-53123-y

**Published:** 2024-01-30

**Authors:** Mohaddeseh Eydi Gabrabad, Mohammadreza Yari, Ziaeddin Bonyadi

**Affiliations:** 1 Student Research Committee, Mashhad University of Medical Sciences, Mashhad, Iran; 2 Department of Environmental Health Engineering, School of Health, Mashhad University of Medical Sciences, Mashhad, Iran

**Keywords:** Biotechnology, Energy science and technology

## Abstract

Microplastics (MPs) are newly recognized contaminants that result from the breakdown of plastics released into aquatic environments. This study focuses on the elimination of polystyrene (PS) using *S. platensis*, a natural biocoagulant, from aqueous solutions. The research investigated several crucial variables, including the initial level of PS ranging from 100 to 900 mg L^−1^, pH levels from 4 to 10, the contact time of 20–40 min, and doses of *S. platensis* ranging from 50 to 250 mg L^−1^. The analysis of the data revealed that the quadratic model offered the best fit for the experimental results. In the present study, we utilized *S. platensis* as a novel natural biocoagulant to effectively eliminate PS from aqueous solutions. Process optimization was performed using a Box–Behnken design (BBD). The best-fitting model for the data was the quadratic model. The results displayed that the highest elimination of PS (81%) was occurred at a pH of 4, with a contact time of 30 min, a dose of *S. platensis* at 250 mg L^−1^, and a PS concentration of 500 mg L^−1^. These findings show that *S. platensis* has a significant effect on removing PS from the aquatic environment. Algae can serve as a convenient and eco-friendly method, replacing chemical coagulants, to effectively remove MPs from the aquatic environment.

## Introduction

Plastic contaminant is a worldwide crisis that presents a serious risk to human health and the environment^1^. Plastics have the potential to degrade under environmental conditions, leading to the formation of tiny particles called MPs. The increase of plastic at both macro and micro levels in the environment has caused many concerns^2^. MPs, measuring less than 5 mm in size, exist in various forms such as spheres, plates, threads, and polyhedral shapes in the environment^3,4^. These pollutants are released into the environment in primary and secondary forms^5^. Primary MPs are intentionally manufactured in small sizes for specific applications such as cosmetics^6^, drug delivery in medicine, and cleaning agents^7^. Secondary MPs are formed as larger plastics break down into smaller particles through physical, chemical, and biological processes^8,9^. Due to their small size, large surface-to-volume ratio, and hydrophobic surface properties, microplastics have a greater tendency to absorb pollutants^10,11^. Hydrophobic organic compounds (HOCs), persistent organic pollutants (POPs), phenanthrene, and antibiotics adsorb onto the surface of MPs and aid in their transportation away from their original sources^12^. These adsorption behavior of MPs can impact crucial biological processes. Some pollutants, such as lead, cadmium^12^, and tetracycline^13^ can be adsorbed onto MPs, ingested by aquatic organisms, and therefore have toxic effects on them. The entrance of these pollutants into the bodies of living organisms may lead to endocrine disorders, which can then affect mobility, reproduction, growth, and enhance the probability of carcinogenesis^14^. Several studies have presented evidence of the extensive presence of MPs in the world's oceans^15,16^. PS is one of the types of MPs, which has the same density as natural water^17^. This specific microplastic contains styrene and benzene monomers, both of which are recognized as carcinogenic and can present a significant danger to both aquatic organisms and humans^18,19^. Microplastic removal has traditionally been conducted through a variety of methods, including chemical, physical, and biological processes^20,21^. In water and wastewater treatment, natural coagulants offer numerous advantages compared to chemical agents. These advantages include biodegradability, minimal toxicity, reduced residual sludge production, and economic feasibility. Compared to chemical coagulants, natural coagulants are a more sustainable and environmentally friendly^22^. Natural coagulants are derived from renewable sources and do not contain harmful chemicals that could have long-term environmental or health effects. Additionally, the utilization of natural coagulants frequently leads to a decrease in residual sludge, thereby reducing both waste production and disposal expenses^23^. Overall, the utilization of natural coagulants can promote a more sustainable approach to water treatment and contribute to a healthier environment^24^. In the recent past, the use of biological approaches, such as algae, has shown potential in addressing this problem. Removing pollutants using biological methods minimizes the formation of toxic byproducts. Ultimately, this approach leads to cleaner ecosystems^25–27^. In some studies such as oak powder^19^, *Aspergillus* sp.^28^, and *S. cerevisiae*^29^ was used to remove MPs from aquatic environment. *S. platensis* is a type of blue-green microalgae that is utilized for the purpose of eliminating pollutants, including heavy metals, organic compounds, and other contaminants, from aquatic environments^30^. Moreover, these algae are abundant in nutrients, minerals, rhamnose sugar, polyunsaturated fatty acids, omega-6, trace elements, and easily digestible enzymes. Even after being used, algal biomass can be utilized as fuel, fertilizer, and medicine to prevent secondary pollution^22^. Owing to its rich nutritional profile and eco-friendliness, the use of algae holds great significance for biotechnological applications^31^. *S. platensis,* being a natural coagulant, assists in the flocculation of MPs and facilitates their effective removal through conventional water treatment processes^32^. Esmaeili et al.^19^ removed polyethylene MPs using *C. vulgaris*. They found that the algae had the maximum elimination rate of 84% in the presence of a pH of 10 and a PE level of 250 mg L^−1^^33^. Also, Cunha et al. (2020^22^) carried out a research by a biopolymer derived from microalgae to remove nanoplastics and MPs from wastewater treatment. They found that the extracellular polymers from algae cause the formation of flocs and effectively remove MPs from the wastewater^22^. During flocculation and coagulation, MPs are brought together by algae, which leads to the formation of larger, denser flocs. This facilitates their effective removal from the water. Generally, the removal of MPs by microalgae is performed through slow and fast agitation stages of the reaction mixture. Finally, the formed flocs settle^34^. In the process of coagulation and flocculation, polysaccharides derived from algae play an important role in creating larger flocs and removing these pollutants by bridging between particles^35^. Biological purification of MPs through algae has attracted scientific attention as a comprehensive and sustainable environmental strategy^32^. Given the mentioned advantages of microalgae, it is important to utilize *S. platensis* for PS removal. To achieve this aim, the removal of PS by *S. platensis* was tested under various conditions, including algae dose, microplastic concentration, and pH.

## Materials and methods

### Chemicals

The polystyrene utilized in the test was in the form of transparent granules were acquired from Pishgaman Plastic Company in Iran.

### *S. platensis* preparation

*Spirulina platensis* (abdf 2224) was sourced from the Iranian National Algae Culture Collection in Tehran, Iran, and grown in Zarrouk’s culture under consistent conditions of 3000 lx light intensity and a temperature of 25 ± 1 °C. Then, the algae cells were centrifuged at 4000 rpm for 10 min and separated from the growth medium, and dehydrated at 80 °C for 12 h.

### PS preparation

Initially, PS granules were ground using a grinder and sifted to sizes smaller than 425 µm. To avoid interfering factors and ensure the contact surface of algae with polystyrene, 0.001 M hydrochloric acid (HCl) is used to wash the raw granules. Then, these materials were dried at 60 °C for 12 h. The resulting MPs were stored in a sealed container, away from any sources of moisture and light, in a dark environment.

### Characteristics and measurements

The properties of S*. platensis*, PS, and biological flocs were examined using FTIR, FESEM, zeta potential, and DLS. FT-IR spectroscopy identified the functional groups present on the surfaces of *S. platensis*, PS, and biological flocs. FESEM investigated the surface morphology of the samples. DLS and ZP analyses were employed to survey the particle size and surface charge of the particles.

### Design of experiments

PS elimination tests were conducted, taking into account various interfering parameters, including the initial PS level (100–900 mg L^−1^), pH range (4–10), and the dose of *S. platensis* (50–250 mg L^−1^). The PS size in all samples was below 425 µm.

The removal process was conducted using the jar test method in two phase. In the first phase, the reaction mixture was prepared according to Table 1 and stirred at 400 rpm for 1 min. In the second stage, the resulting suspension was mixed at a speed of 100 rpm for 15 min. The suspension was then conveyed to a decanter funnel, and the resulting flocs were settled for 30 min without any disturbance. After an appropriate settling period, the liquid above the settled particles was collected, filtered, dehydrated at 55 °C for 12 h, and then weighed. The elimination rate of PS (R %) was calculated using Eq. (1):

**Table 1:** Main factors utilized to eliminate PS.

Factor	Code	Value		
		− 1	0	+ 1
PS (mg L^−1^)	A	100	500	900
*S. platensis* (mg L^−1^)	B	50	150	250
pH	C	4	7	10

1 $$\mathrm{R }\left(\mathrm{\%}\right)= \frac{{{\text{M}}}_{1}-{{\text{M}}}_{2}}{{{\text{M}}}_{1}}\times 100\mathrm{\%}$$

M1 denotes the original weight of PS before the elimination process, while M2 signifies the weight of PS on a Whatman filter subsequent to the elimination.

### Modeling PS removal

In this work. It was employed the BBD model to improve the efficiency of eliminating PS MPs. The RSM quadratic model can be expressed as Eq. (2) below:

2 $${\text{Y}}=\upbeta 0+{\sum }_{i=1}^{k}{\beta }_{i}{x}_{i}+\sum_{i=1}^{k}{\beta }_{ii}{x}_{i}^{2}+\sum_{1\le i\le j}^{k}{\beta }_{ij}{x}_{i}{x}_{j}$$

The Eq. (1) uses various symbols to represent different factors. Y stands for the anticipated outcome, β0 represents the fixed coefficient, βi represents the coefficient for linear effects, βii represents the quadratic coefficient, βij represents the coefficient for interaction, and xi or xj denote the coded values of the factors^36^.

## Result and discussion

### Characterization

**Figure 1:**
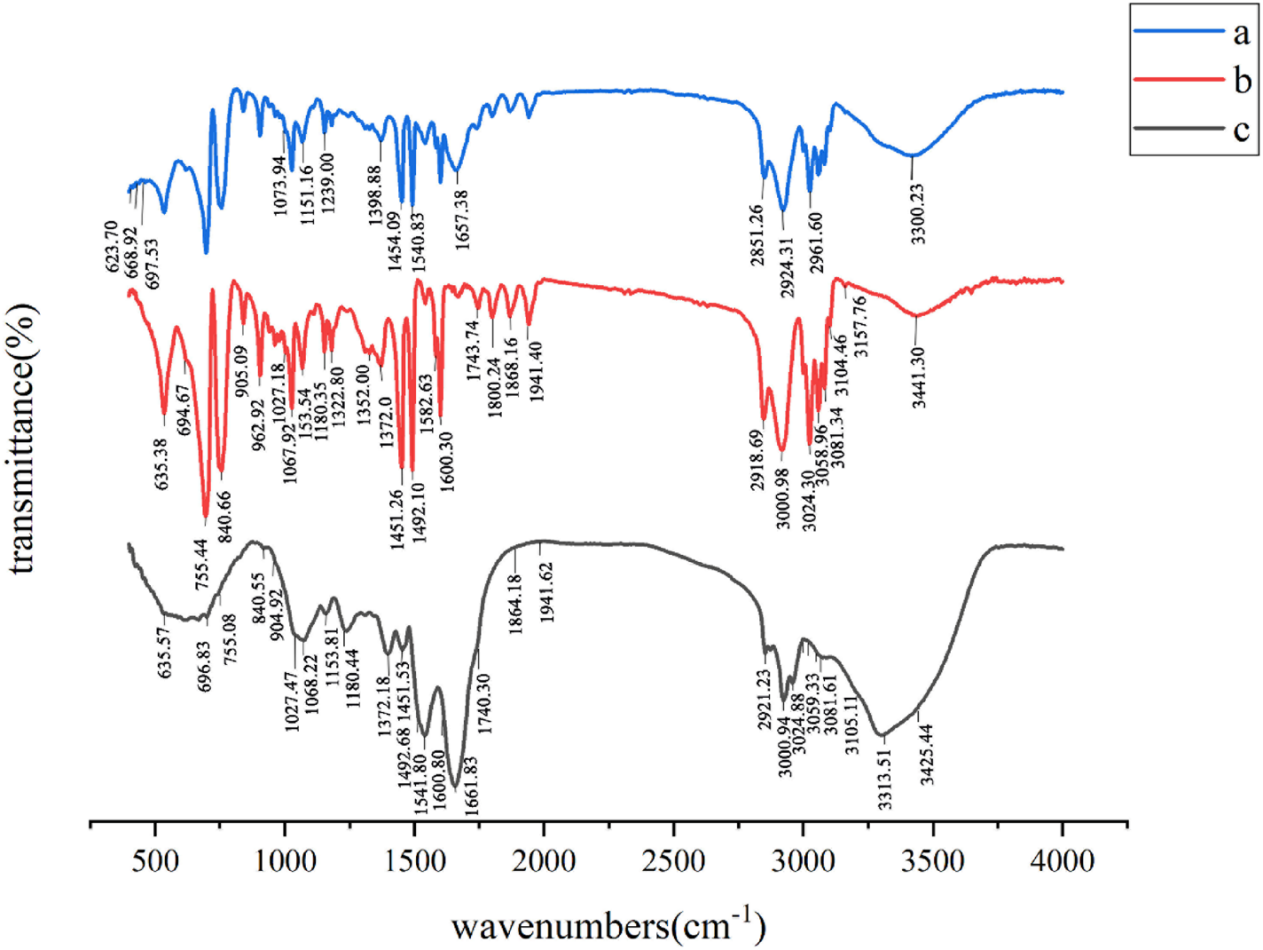
FT-IR spectrum of (**a**) *S. platensis*, (**b**) PS, and (**c**) biological flocs.

Figure 1 indicates FT-IR spectrum of *S. platensis*, PS, and biological flocs. The analysis confirmed that the formed flocs contained PS. From Fig. 1a, the adsorption peaks at 6238.51 cm^−1^, 668 cm^−1^, and 697 cm^−1^ show the presence of alkyl halides. The adsorption bands at 1073–1398 cm^−1^ correspond to the stretching vibration of the CO–H group in polysaccharides, including uric acid. Nucleic acid spectra were discovered at 1239 cm^−1^. The proteins exhibited bending in the CH_2_ and CH_3_ groups. The peak of 1398 cm^−1^ was related to the (NCH_3_)_3_ groups^37^. The specific peak observed at 1454 cm^−1^ corresponds to the vibration of the C–N bond, which is specifically found in the quaternary ammonium groups. The peak detected at 2851 cm^−1^ is associated with the C–H stretching vibration characteristic of fatty acids^38^. The peak observed at 3300 cm^−1^1 is linked to the stretching vibration of the O–H bond, which can be attributed to lignocellulose and water molecules. Additionally, the peak at 3300 cm^−1^ corresponds to the vibration of the N–H bond in the primary amine group, indicating the presence of protein within the algae's structure. These proteins have the ability to create connections between PS particles, causing the formation of relatively large flocs. Consequently, these flocs tend to settle down due to their increased size and weight^39^. Based on the information provided in Fig. 1b, the peaks detected at 635.38 cm^−1^ and 694 cm^−1^ can be attributed to the bending vibration of the C–H groups. Furthermore, the peak observed at 755.44 cm^−1^ may be associated with the ring-bending vibration of the polystyrene structure. The absorption peaks at 1067.92 cm^−1^ and 1372 cm^−1^ are indicative of the bending vibrations of the C–H bonds. The peaks at 1492.1 cm^−1^ and 1600.30 cm^−1^ correspond to the C=C stretching vibration, which is a characteristic feature of the aromatic ring structure present in polystyrene. Lastly, the peaks registered at 2918.69 cm^−1^ and 3000.98 cm^−1^ suggest the stretching vibrations of the C–H bonds within the polymer^40,41^.

Figure 1c provides evidence of the presence of biological flocs, highlighting the connection between the functional groups and PS. Upon the absorption of PS, specific absorption bands associated with N–H, C–H, C=O, and CO–H functional groups underwent shifts to 3425 cm^−1^, 3313 cm^−1^, 1864 cm^−1^, and 1064 cm^−1^, respectively. These shifts indicate the interaction and linkage between PS and *S. platensis*. The peak observed at 1661 cm^−1^ corresponds to the stretching vibration of the C=O bond in carboxyl and carboxylic groups, which play a role in the adsorption and bridging mechanism between PS particles.

**Figure 2:**
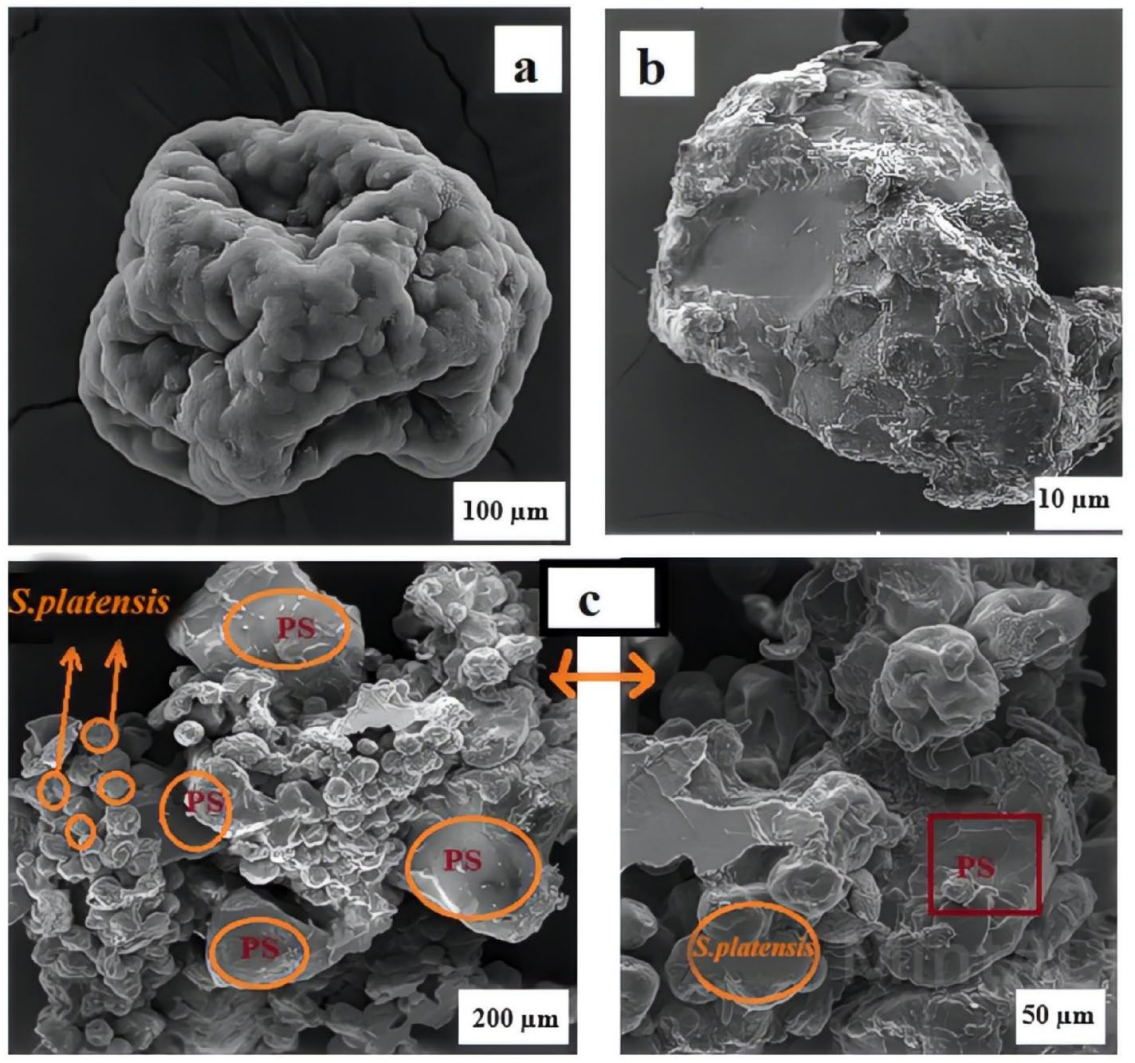
FESEM of (**a**) *S. platensis*, (**b**) PS, and (**c**) the biological flocs of *S. platensis* and PS.

Figure 2a–c displays the FESEM images of *S. platensis* and PS. As shown in Fig. 2a, S*. platensis* is found in the form of spherical particles with heterogeneous and non-uniform pores. This characteristic of microalgae allows it to attach to PS particles^42^. Figure 2b, in contrast, displays crushed PS particles that exhibit irregular cracks on their surfaces. This characteristic results in an increased contact area with algal particles^43^. Figure 2c shows the flocs that contain microalgae and MPs after the elimination process. The entanglement and accumulation of MPs and algae are clearly visible, indicating the adsorption and bridging of MPs by algae cells^44^. The interaction between the pores and gaps found in algae particles and the uneven surface of MPs leads to the formation of large flocs. Consequently, these flocs settle rapidly due to their increased size and weight^45^. In conclusion, these findings confirm that *S. platensis* induces the coagulation and removal of PS.

The zeta potential (ZP) measures the electrostatic dispersion process and provides an indication of the relative stability of particles. It is influenced by various parameters, including pH, solution conductivity, and particle concentration (37). Table 2 presents the ZP values for PS and biological flocs^46^. According to Table 2, it is found that the initial ZP value for PS particles was − 76.3 mV, which decreased to − 46.9 mV after the formation of biological flocs composed of PS and algae. This indicates that the addition of algae to the suspension reduces the repulsive force between particles, thereby promoting the formation of biological flocs. Over time, these flocs settle due to their increased density, indicating the potential of algae to influence the settling and removal of PS particles from the environment.

**Table 2:** Zeta potential and DLS levels for both PS and biological flocs.

Material	Zeta potential (mV)	Average diameter (µm)
PS	− 76.3	328.40
Biological floc	− 46.9	673.7

According to Table 2, the results of the DLS analysis indicate significant changes in the particle size of both PS particles and biological flocs. The data demonstrates that the average diameter (D_avg)_ of PS increased following elimination, suggesting the formation of biological flocs that incorporate PS particles. Initially, the D_avg_ of PS was measured at 328.4 μm. However, after the removal process, the D_avg_ of the biological flocs containing PS particles rose to 673.7 μm. These observations indicate that coagulation and flocculation mechanisms effectively remove PS particles from the aqueous medium. Due to the presence of polysaccharides and proteins in their cell walls, algae can facilitate the formation of biological clots by acting as a bridge between PS particles^47^.

### Response model

The BBD statistical technique is utilized to design, model, and optimize the elimination of PS. BBD employs a three-level quadratic design, examining variables at the midpoint (± 1) and center point (0)^48^. In this study, a total of 17 experimental trials were conducted using BBD to investigate the effects of four process factors: dosage of *S. platensis*, pH, and initial PS concentration. Table 3 presents the elimination rate of PS through the use of *S. platensis*.

**Table 3:** BBD matrix used for the elimination of PS by *S. platensis.*

Run No	Coded variable			Removal (%)	Run No	Coded variable			Removal (%)
	A	B	C			A	B	C	
1	100	150	10	52	10	500	50	4	39.68
2	500	150	7	63.6	11	500	250	4	81
3	100	50	7	19.6	12	500	50	10	51.68
4	900	50	7	53	13	500	150	7	69
5	500	150	7	78	14	500	150	7	70
6	500	150	7	80	15	900	250	7	49.8
**7**	500	250	10	60.16	16	100	150	4	41.8
8	900	150	4	62	17	900	150	10	55.4
9	100	250	7	54					

Based on Table 3, the PS elimination rates varied between 19.6 and 81%. This study aimed to survey the impact of various independent factors, such as initial PS level, *S. platensis* dose, pH, and contact time, on the rate of PS elimination. The researchers analyzed the experimental results using various models, such as linear, 2FI, quadratic, and cubic models, to identify the most suitable model for accurately representing the findings. The statistical adequacy assessment of these models is presented in Table 4.

**Table 4:** Statistical adequacy assessment of models.

Source	Sequential p value	Lack of fit p value	Adjusted R^2^	Predicted R^2^
Linear	0.1454	0.1113	0.1751	− 0.2118
2FI	0.0662	0.2035	0.4607	0.0630
Quadratic	0.0295	0.6916	0.7704	0.4367
Cubic	0.6916		0.7108	

Table 5 illustrates the coefficients of the quadratic model (QM) for the elimination of PS by *S. platensis*. It also demonstrates the goodness of fit of QM to the findings.

**Table 5:** Estimated coefficients for the QM of PS elimination by *S. platensis*.

Factor	Coefficient estimate	df	Standard error	95% CI low	95% CI high	VIF
Intercept	70.92	1	3.53	62.56	79.28	
A-Conc	0.4750	1	2.79	− 6.13	7.08	1
B-Dose	13.38	1	2.79	6.77	19.98	1
C-pH	0.4700	1	2.79	− 6.14	7.08	1
AB	− 14.90	1	3.95	− 24.24	− 5.56	1
AC	− 7.45	1	3.95	− 16.79	1.89	1
BC	− 9.21	1	3.95	− 18.55	0.1312	1
A^2^	− 10.45	1	3.85	− 19.55	− 1.35	1.01
B^2^	− 10.87	1	3.85	− 19.97	− 1.77	1.01
C^2^	− 0.9200	1	3.85	− 10.02	8.18	1.01

Equation (3), derived from the information in Table 5, provides a quantitative model for PS elimination by utilizing the coded factors. The equation can be expressed as follows:

3 $${\text{Elimination }}\% = {7}0.{92} + 0.{475}0{\text{A}} + {13}.{\text{38B}}{-}0.{\text{47C}} - {14}.{\text{9AB}}{-}{7}.{\text{45AC}}{-}{9}.{\text{21BC}}{-}{1}0.{\text{45A}}^{{2}} {-}{1}0.{\text{87B}}^{{2}} {-}0.{\text{92C}}^{{2}}$$

The models used in the study consisted of fixed and variable components, considering different laboratory factors. The predicted elimination rate obtained from the model was 70.92%. The coded variables A, B, and C had coefficients of 0.4750, 13.38, and 0.47, respectively. Among these factors, the dose of *S. platensis* (coded as B) had the most significant impact on the elimination rate, with a coefficient of 13.38. The interaction effect of AB had the highest coefficient of 14.90, while the squared effect of B^2^ had the highest coefficient of 10.87. The analysis of variance (ANOVA) for the quantitative model of PS elimination by *S. platensis* is presented in Table 6.

**Table 6:** The ANOVA results for the quantitative model of PS elimination by *S. platensi.*

	Sum of squares	df	Mean square	F value	p value
Model	3741.95	9	415.77	11.64	0.0019
A-Conc	348.48	1	348.48	9.76	0.0168
B-Dose	820.13	1	820.13	22.96	0.0020
C-pH	3.43	1	3.43	0.0961	0.7656
AB	353.44	1	353.44	9.90	0.0162
AC	70.56	1	70.56	1.98	0.2026
BC	269.62	1	269.62	7.55	0.0286
A^2^	1170.76	1	1170.76	32.78	0.0007
B^2^	541.93	1	541.93	15.17	0.0059
C^2^	29.46	1	29.46	0.8248	0.3940
Residual	250.00	7	35.71		
Lack of fit	66.51	3	22.17	0.4833	0.7117
Pure error	183.49	4	45.87		
Cor total	3991.95	16			
R^2^	0.93		Predicted R^2^	0.66	
Adjusted R^2^	0.85		Adeq precision	12.90	

**Figure 3:**
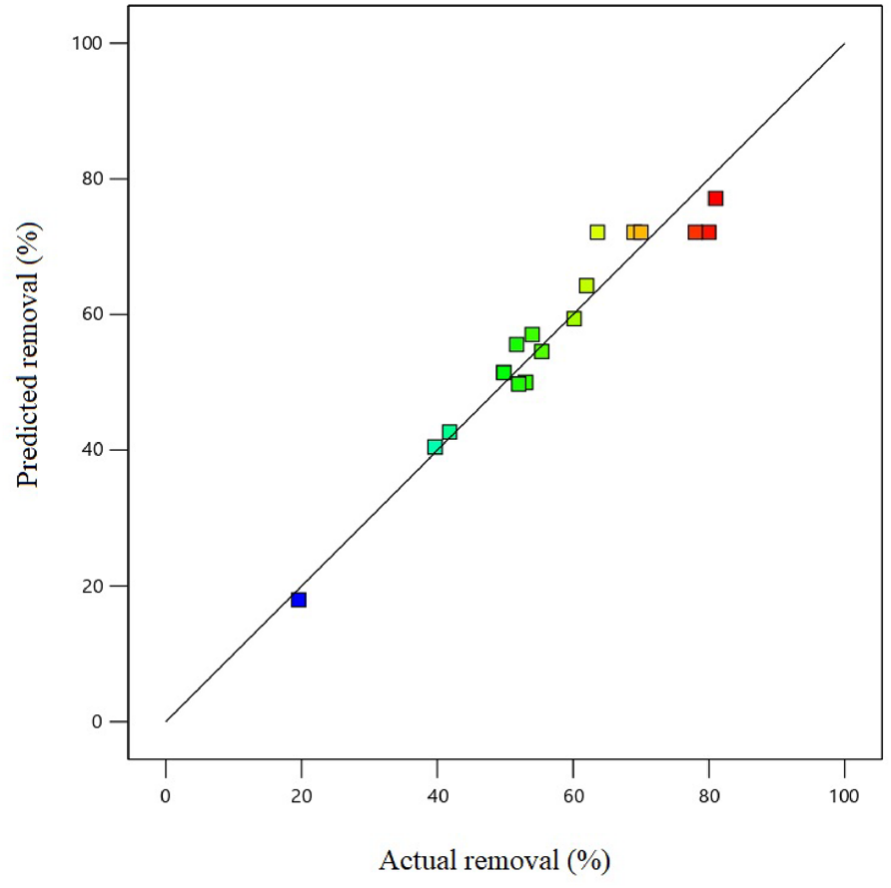
Graph of predicted removal against actual removal.

Figure 3 demonstrates the comparison between the predicted removal and the actual removal of PS. It is apparent from Fig. 3 that the model accurately predicts the PS removal.

### Impact of main variabls on elimination efficiency

**Figure 4:**
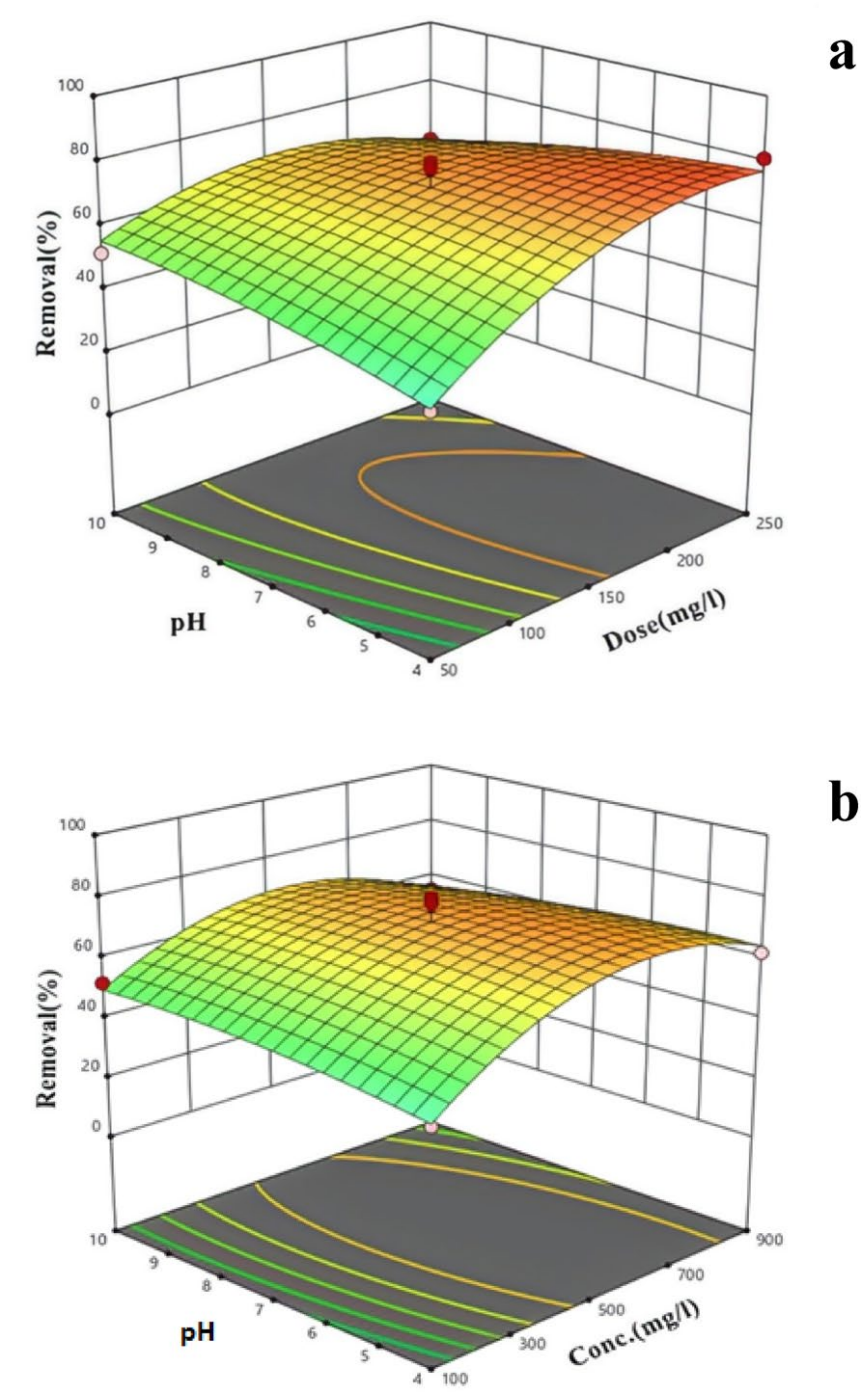
The 3D surface graph and contour plots for PS removal as a function of (**a**) pH and *S. platensis* dose; (**b**) pH and PS concentration.

Figure 4a,b illustrates the relationship between the elimination of PS and various variable, such as initial PS level, contact time, pH, and *S. platensis* dose. The graph illustrates how changes in microalgae dose, pH, PS concentration, and contact time affect the efficiency of PS elimination.

The findings from Fig. 4a indicate that changes in pH did not have a important effect on the elimination efficiency of PS (P value > 0.05). However, it is noteworthy that the maximum elimination efficiency was observed at a pH of 4. One possible explanation for the increased elimination of MPs at pH 4 is the favorable conditions for interaction between MPs and microalgae. At a pH of 4, there is a slight prevalence of positively charged groups in the algal structure compared to the negatively charged groups. This dominance facilitates the adhesion and connection between algal cells and negatively charged PS particles. Consequently, this improved interaction facilitates the efficient accumulation of MPs and contributes to an increased rate of elimination^49^.

Based on the results of Fig. 4a, increasing the microalgae dose from 50 to 250 mg L^−1^ resulted in a 28.57% increase in PS elimination efficiency (p value < 0.05). This is because as the algal dose increases, the probability of algal cells being exposed to PS particles also increases. This means that the number of algae binding sites increases, facilitating the bridging between PS particles and algae. This ultimately results in the dense floc production, which can settle quickly over time. This mechanism ultimately leads to the efficient elimination of PS particles from aqueous solution^47^.

Figure 4b clearly depicts the correlation between increasing PS concentration (from 100 to 900 mg L^−1^) and the corresponding rise in PS elimination efficiency (from 39 to 70%). This increase in elimination efficiency is statistically significant (P value < 0.05). A higher level of PS particles enhances the likelihood of contact between PS MPs and algal cells during the elimination process, providing more opportunities for interaction and binding. The attachment of PS particles to algal cells is a critical step in the elimination process and is facilitated by electrostatic interactions between the positively charged PS molecules and the negatively charged cell surface of the algae. This interaction leads to the formation of flocs, which can be easily separated from the water, resulting in effective elimination of MPs from the aqueous solution^50^. According to Yu-Ru et al.^51^, the elimination rate of microplastics (MPs) is directly correlated with their concentration. Yuanyuan et al.^52^ demonstrated that microalgae exhibit higher MPs elimination rates when exposed to elevated levels (e.g., 1 g L^−1^) compared to lower levels (e.g., 0.01, 0.1 g L^−1^). In another investigation, bacterial biofilms achieved an 84.5% elimination of PS MPs at a level of 10 mg L^−1^^53^.

## Future prospective

We propose implementing waste-to-energy (WtE) technology, specifically pyrolysis, as a promising method for disposing of PS. Pyrolysis transforms PS waste into valuable products, such as oils and gases, while minimizing environmental impacts. By incorporating pyrolysis into the waste management process, we can effectively address the issue of PS disposal, contributing to a more sustainable approach while simultaneously harnessing energy from the process. This indicates a promising direction for future research and development in the field of PS waste management.

## Conclusion

In this study, we employed *S. platensis* as a new natural biocoagulant for the efficient elimination of PS from water solutions. The quadratic model proved to be the best fit for the data. The findings indicated that the maximum PS elimination (81%) was attained at a pH of 4, a contact time of 30 min, a *S. platensis* dose of 250 mg L^−1^, and a PS level of 500 mg L^−1^. These results demonstrate the significant impact of *S. platensis* on PS elimination in water environments. Microalgae present a practical and environmentally friendly alternative to chemical coagulants for the effective elimination of MPs from aquatic settings.

## Data Availability

The datasets generated and analyzed during the current study were available from the corresponding author on reasonable request.
